# Altered Connectivity of the Frontoparietal Network During Attention Processing in Prolactinomas

**DOI:** 10.3389/fneur.2021.638851

**Published:** 2021-08-30

**Authors:** Chenglong Cao, Yu Wang, Jia Liu, Aobo Chen, Jinjiang Lu, Guozheng Xu, Jian Song

**Affiliations:** ^1^Department of Neurosurgery, The First School of Clinical Medicine, Southern Medical University, Guangzhou, China; ^2^Department of Cognitive Neuroscience, Faculty of Psychology and Neuroscience, Maastricht University, Maastricht, Netherlands; ^3^The Key Laboratory of Biomedical Information Engineering of Ministry of Education, Institute of Biomedical Engineering, School of Life Science and Technology, Xi'an Jiaotong University, Xi'an, China; ^4^Foreign Linguistics and Applied Linguistics, Research Institute of Foreign Languages, Beijing Foreign Studies University, Beijing, China; ^5^Department of Neurosurgery, The General Hospital of Chinese PLA Central Theater Command, Wuhan, China

**Keywords:** prolactinomas, attention processing, prolactin, theta coherence, alpha coherence

## Abstract

Prolactinomas have been reported for the failure of cognitive functions. However, the electrophysiological mechanisms of attention processing in prolactinomas remain unclear. In a visual mission, we monitored the scalp electroencephalography (EEG) of the participants. Compared with the healthy controls (HCs), larger frontoparietal theta and alpha coherence were found in the patients, especially in the right-lateralized hemisphere, which indicated a deficit in attention processing. Moreover, the frontoparietal coherence was positively correlated with altered prolactin (PRL) levels, implying the significance of PRL for adaptive brain compensation in prolactinomas. Taken together, this research showed the variations in attention processing between the HCs and prolactinomas. The coherence between frontal and parietal regions may be one of the possible electrophysiological biomarkers for detecting deficient attention processing in prolactinomas.

## Introduction

Pituitary adenomas are the second most common intracranial tumors, accounting for about 16.5% of the central nervous system (CNS) tumors (1). The mechanical pressure from tumor mass on adjacent neuroanatomical regions (e.g., the inferior frontal lobe, diencephalon, optic chiasma, pituitary stalk, etc.) could disrupt the tissue structures (2, 3) and then decrease the endocrine functions of the hypothalamus or pituitary stalk. Apart from the physical damages, the functioning pituitary adenoma may abnormally secrete high hormone levels and, thus, impair cognition functions in these patients with pituitary adenomas (4), leading to less social contact and lower living quality (5). Although the underlying pathophysiology has not been completely understood, electrophysiological researches suggest that endogenous hormone abnormalities contribute to cognitive impairments in executive performance (6, 7), pre-attention (8), processing speed (9), and working memory (10). Electrophysiological research has manifested that altered levels of blood hormones can affect brain structure in pituitary patients with Cushing syndrome, acromegaly, and prolactinomas (11–13). These researches above strongly demonstrate that disturbances in endogenous hormones could harm the brain structure and then likely lead to corresponding brain dysfunctions. Specifically, pituitary patients frequently suffer from the dysfunction in attention processing leading to a decline in the quality of life of the patients (14, 15). However, to date, no electrophysiological studies of frontoparietal networks in attention processing have been conducted on prolactinomas, which will be discussed in the present study by analyzing the event-related phase coherence (ERPCoh).

P200, whose peak latency ranges from 100 to 200 ms, is sensitive to emotional stimuli and reflects the attention bias occurring automatically (16, 17). Hence, P200 can be regarded as an attention-related component (18). While P200 amplitudes predominantly reflect the amount of synchronous activity in the local region, the degree of interactions between two electrode pairs can be measured by coherence (19). Previous research found that functional connectivity can be regarded as a well-established biomarker for cognitive impairments (20). Long-range communications appeared to be mediated by lower-frequency oscillations, especially the alpha and theta frequency ranges, which, in general, appear to fulfill a messenger function (21–23). Alpha phase interactions between distinct regions directly supported the neuronal processing underlying attentional function (24). Theta coherence between the frontal and parietal region would be strong if task-relevant stimuli were processed (25). This also indicated that theta coherence might have a pivotal role in attention tasks, especially in the right frontoparietal regions (26). Previous research revealed several significant group differences in power and coherence values (27). Concussed participants showed enhanced coherence in the low-frequency bandwidths between several regions of interest. Increased coherence meant that concussed participants were able to recruit additional brain networks to compensate for the impaired cognition. Furthermore, this altered intrinsic coherence is currently under investigation as a potential imaging biomarker in a variety of psychiatric disorders (27, 28).

Attention processing has been associated with frontal and parietal regions. The bilateral superior longitudinal fasciculus (SLF) connects the frontal regions with the parietal regions, and it consists of three frontoparietal longitudinal pathways (29–31). The SLF I is the most dorsal pathway and connects the precuneus and superior parietal lobule to the superior frontal lobes. The SLF III is the most ventral pathway and extends from the temporoparietal junction/supramarginal gyrus to the inferior frontal lobes. The SLF II, overlapping with the prefrontal component of the dorsal network and the parietal component of the ventral network, directly connect the dorsal networks with ventral networks.

In the current study, we aimed to examine whether the attention network was impaired by comparing the performances of prolactinomas to those of the healthy participants. ERPCoh was used for analyzing the attention network in a passive viewing paradigm in prolactinomas. We hypothesize that prolactinomas would show abnormal frontoparietal networks. Accordingly, we further predicted that the altered intrinsic coherence within these frontoparietal networks related to attention processing may be associated with the abnormal serum prolactin levels in prolactinomas.

## Methods

### Participants

Prolactinomas were recruited in the Department of Neurosurgery, Wuhan School of Clinical Medicine, Southern Medical University (China). This prospective study was approved by the ethics committee of Wuhan School of Clinical Medicine, Southern Medical University. The written informed consent was fully understood and signed by all participants. The inclusion and exclusion criteria were described in our previous work (32). Patients were included if (1) a prolactin-secreting pituitary tumor was discovered (33, 34), (2) they had never undergone a craniotomy or received radiation therapy, (3) they were able to fulfill ERP examinations, and (4) their age ranged from 20 to 50 years old, with all of them completing at least secondary school education. Patients were excluded if they (1) had a history of neurologic or psychiatric disorders; (2) had comorbidities that could affect cognitive function, including severe liver, hypertension, heart, or kidney dysfunctions; (3) had severe complications, such as coma, infection, epilepsy, hydrocephalus, and leaking of cerebrospinal fluid; and (4) had drug or alcohol abuse [subjects who drink alcohol over 2.0 standard drinks (10 g of pure alcohol) during the day and meet any 2 of the 11 criteria under the DSM-V in the past year] (35) or were on any medications (including oral contraceptives). In this research, the volume of the tumor may have a fundamental effect on our observations because reports have demonstrated that macroadenomas can affect the brain structure (36, 37). The population sample in this research was strictly chosen to rule out big tumors that compress the surrounding neuroanatomical structures (see representative brain images of a few patients regarded in the Supplementary Materials).

Twenty prolactinoma patients and 20 healthy controls (HCs) were recruited to perform the assigned task. Their age [*t*_(40)_ = 0.933, *p* = 0.340], gender (χ^2^ = 0.404, *p* = 0.525), and education [*t*_(40)_ = 0.207, *p* = 0.652] were matched between these two groups (see Table 1).

**Table 1:** Demographic and clinical characteristics: prolactinoma patients and healthy controls (HCs).

	**Patients**	**HCs**	***p*** **-value**
	**(***n*** = 20)**	**(***n*** = 20)**	
Age (years)	34.75 (30.00–40.00)	33.95 (29.00–38.00)	0.340^a^
Gender Male	10 (50%)	8 (40%)	χ2 = 0.404
Female	10 (50%)	12 (60%)	0.525^b^
Education (years)	12.35 (9.00–16.00)	12.70 (9.00–16.00)	0.652^a^

*Data are presented as mean values and ranges (minimum and maximum values)*.

a *Two independent samples t-test*.

b *Chi-square tests*.

### Stimuli and Procedure

**Figure 1:**
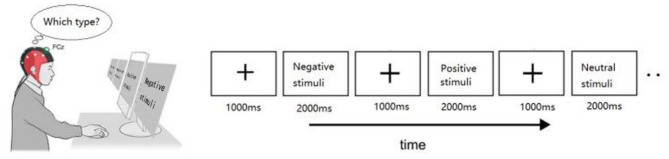
Illustration of the stimulus paradigm applied.

The IAPS (International Affective Picture System) provides basic and reliable experimental materials for studies on visual processing (38). The valence and arousal of the pictures in the IAPS have been rated. These ratings have been established to be robust and reliable over thousands of ratings using several scales, primarily the self-assessment manikin (39). In our experiment, 45 pictures were selected from the IAPS, comprising affective stimuli (including positive and negative images) and neutral stimuli, 15 images for each stimulus type. E-prime (Psychology Software Tools) was used to regulate the stimulus presentation. The trial started with a white fixation mark (+) for 1,000 ms on the black screen, and then a random picture was displayed for 2,000 ms (see Figure 1). The materials were displayed in a pseudorandom order, and a particular stimulus appeared less than four times consecutively. During the experiment, participants sat in the semi-dark test room with the screen 100 cm away from the eyes. Moreover, participants were instructed to view these stimuli and then determine whether the stimulus was positive, negative, or neutral in mind. For valence and arousal dimensions, a one-way ANOVA was measured. *Post-hoc* test contrasts revealed that there were no distinct differences in arousal between positive and negative stimuli. Besides, there were significant differences between affective stimuli and neutral stimuli in both arousal and valence (see Supplementary Table 1).

### Electroencephalography Recording

Raw EEG was recorded using a 64-Ag/-AgCl electrode cap based on the international 10–10 system and an EEG amplifier (all from eego^TM^). The EEG signal was amplified at a sampling rate of 1,000 Hz. All electrode impedances were kept below 5 kΩ.

### Electroencephalography Analysis

The raw EEG data were preprocessed by using the EEGLAB toolbox (40). First, we re-referenced the EEG data to the averaged mastoids (M1 and M2) and bandpass filtered the data to 1–49 Hz. Second, we applied SASICA (a plugin from EEGLAB) to correct the blink artifact (7, 41). Finally, we segmented the continuous EEG into the epoch from 350-ms pre-stimulus to 1,200-ms post-stimulus and corrected the baseline to the mean amplitude of the pre-stimulus interval. In this paper, the analysis focused mainly on the P200 component, and the time windows for investigating P200 is 180–280 ms.

The event-related phase coherence (ERPCoh) was used to measure the degree of synchronization between two channels across time in the specific frequency band and is related to the communication between different brain regions (40).

$$\begin{array}{l} ERPCoh^{a,b}\left(f,t\right)=\frac{1}{n}\overset{n}{\underset{k=1}{\sum }}\frac{F_{k}^{a}\left(f,t\right)F_{k}^{a}{\left(f,t\right)}^{*}}{\left|F_{k}^{a}\left(f,t\right)F_{k}^{b}\left(f,t\right)\right|} \end{array}$$

*F*_*k*_*(f,t)* is the spectral estimate of trial *k* at frequency *f* and time *t* by using a sinusoidal wavelet, whereas *F*_*k*_(f,t)^*^ is the complex conjugate of *F*_*k*_*(f,t)*. We focused on the theta (4–8 Hz) and alpha (8–12 Hz). ERPCoh is gotten by the newtime *f* () function from EEGLAB toolbox using Morlet wavelets in a 4- to 30-Hz frequency band (from two cycles at the lowest frequency to six cycles at the highest; the frequency is 4–30 Hz, and the number of output time is 100).

**Figure 2:**
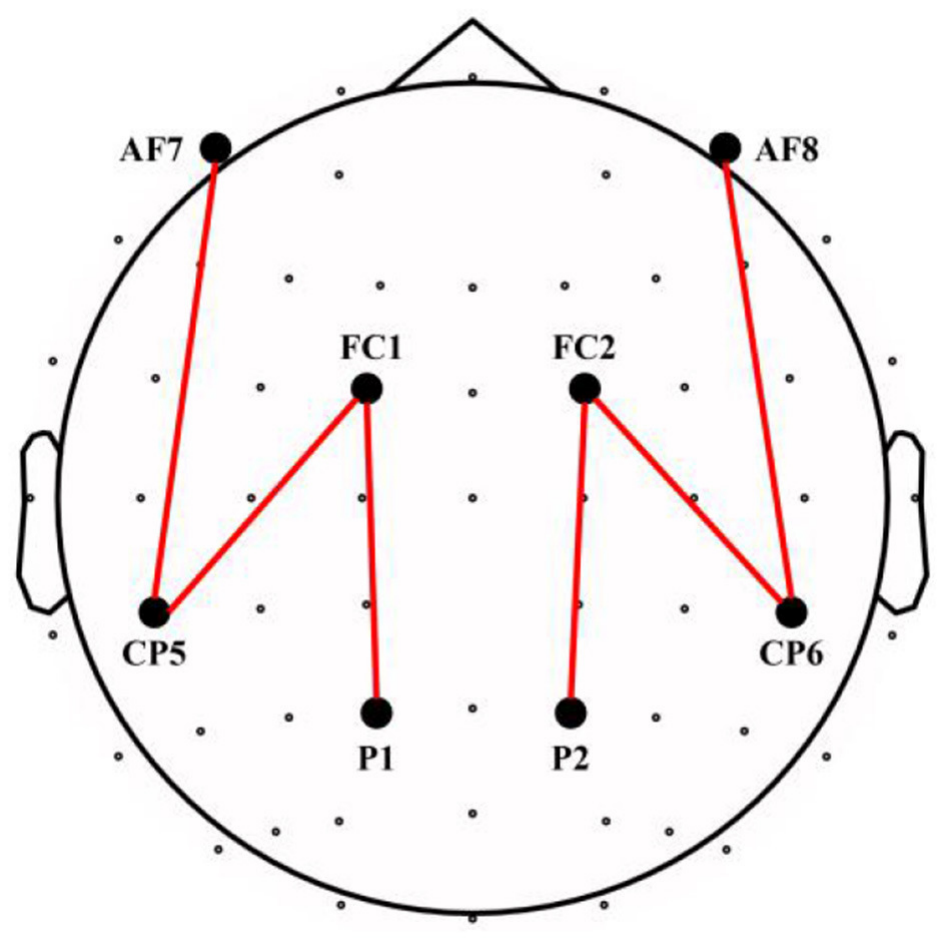
Electrode pairs for the frontoparietal network.

Koessler et al. (42) demonstrated the automated cortical projection of EEG sensors. In that, the mean cortical projections of FC1 and FC2 are on the superior frontal gyrus, and AF7 and F8 are on the middle frontal gyrus. Similarly, P1 and P2 correspond to the precuneus, and CP5 and CP6 correspond to the supramarginal gyrus. To quantify the ability of attention processing, we extracted theta and alpha ERPCoh at the frontoparietal network. In that, the mean cortical projections of FC1–P1 and FC2–P2 electrode pairs are on the dorsal frontoparietal network, and the AF7–CP5 and AF8–CP6 electrode pairs correspond to the ventral frontoparietal network. The FC1–CP5 and FC2–CP6 electrode pairs are projected on communication between the dorsal and ventral networks (see Figure 2).

### Correlation Analysis

According to the conclusion of former research, an abnormally high prolactin (PRL) level has a quite important influence on cognitive functions, so that the Pearson correlation coefficient between PRL level and ERPCoh was measured in patients.

### Statistical Analysis

The homogeneity and normality test of variance was applied in the frontoparietal theta and alpha coherence within the P200 time window. To evaluate the difference of frontoparietal coherence between patients and HCs, two-samples *t*-test was used in the theta and alpha band ERPCoh. In addition, as multiple comparisons were used, all *p*-values were corrected by false discovery rate (FDR <0.05) to control for false positives.

## Results

### Event-Related Potentials

**Figure 3:**
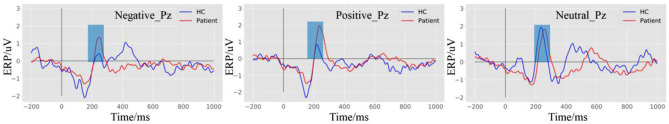
Event-related potential (ERP) waveforms elicited by negative, positive, and neutral stimulus at the Pz electrode.

Grand average ERPs elicited by the negative, positive, and neutral stimuli for the patients and HCs are presented in Figure 3. Under the negative and the positive targets, we observed a significant increase in the P200 peak of patients compared with HCs.

### Event-Related Phase Coherence

**Figure 4:**
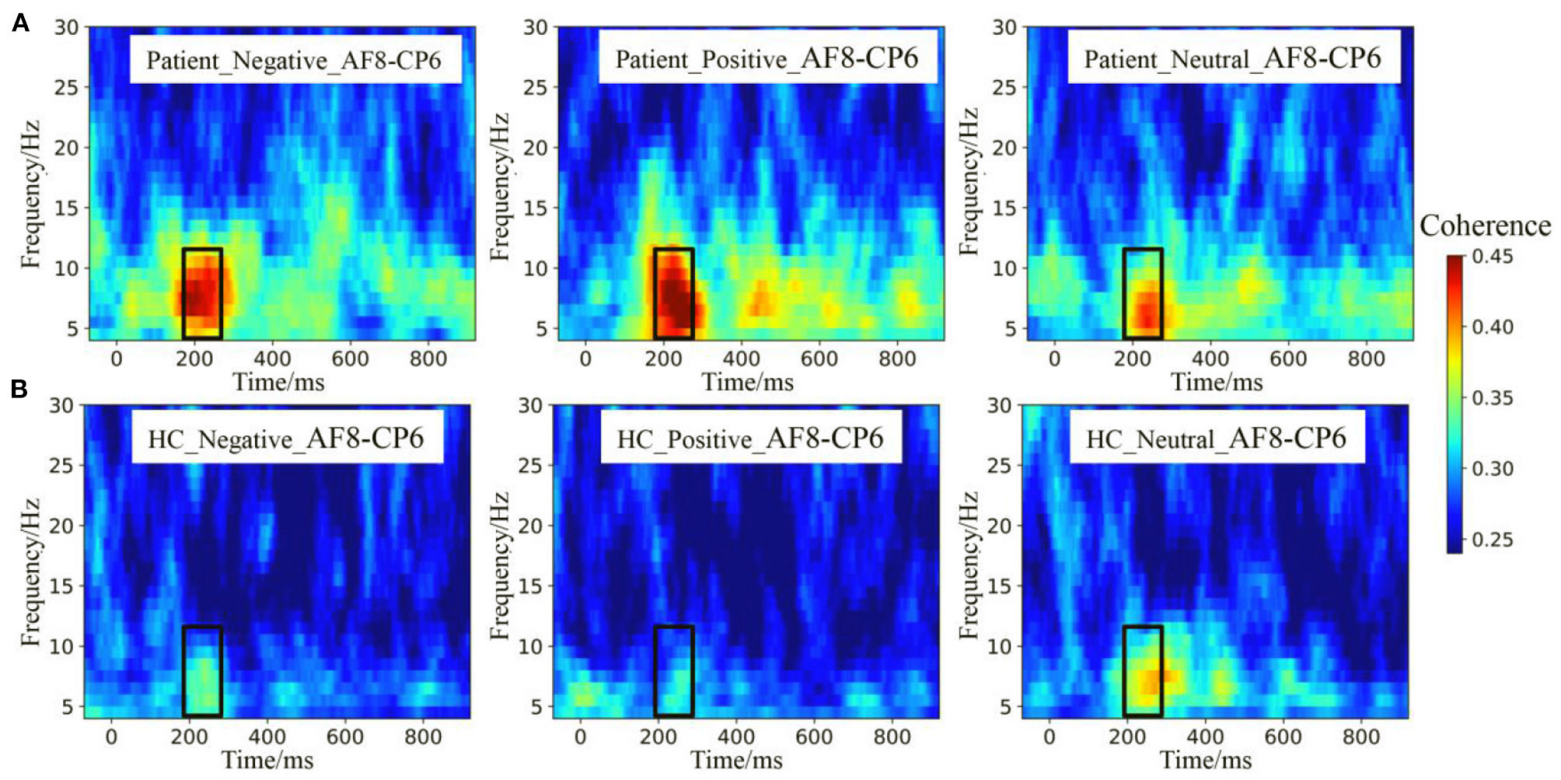
Event-related phase coherence (ERPCoh) between the frontal and parietal electrodes. **(A)** The theta and alpha band ERPCoh from the AF8–CP6 electrode pairs in patients. **(B)** The theta and alpha band ERPCoh from the AF8–CP6 electrode pairs in HCs.

The theta and alpha band ERPCoh from the AF8–CP6 electrode pairs are depicted in Figure 4. Visual inspection showed that ERPCoh was significant within 180–280 ms for different stimuli across all participants. Moreover, the theta and alpha ERPCoh in the patients were stronger than in HCs between 180 and 280 ms.

**Figure 5:**
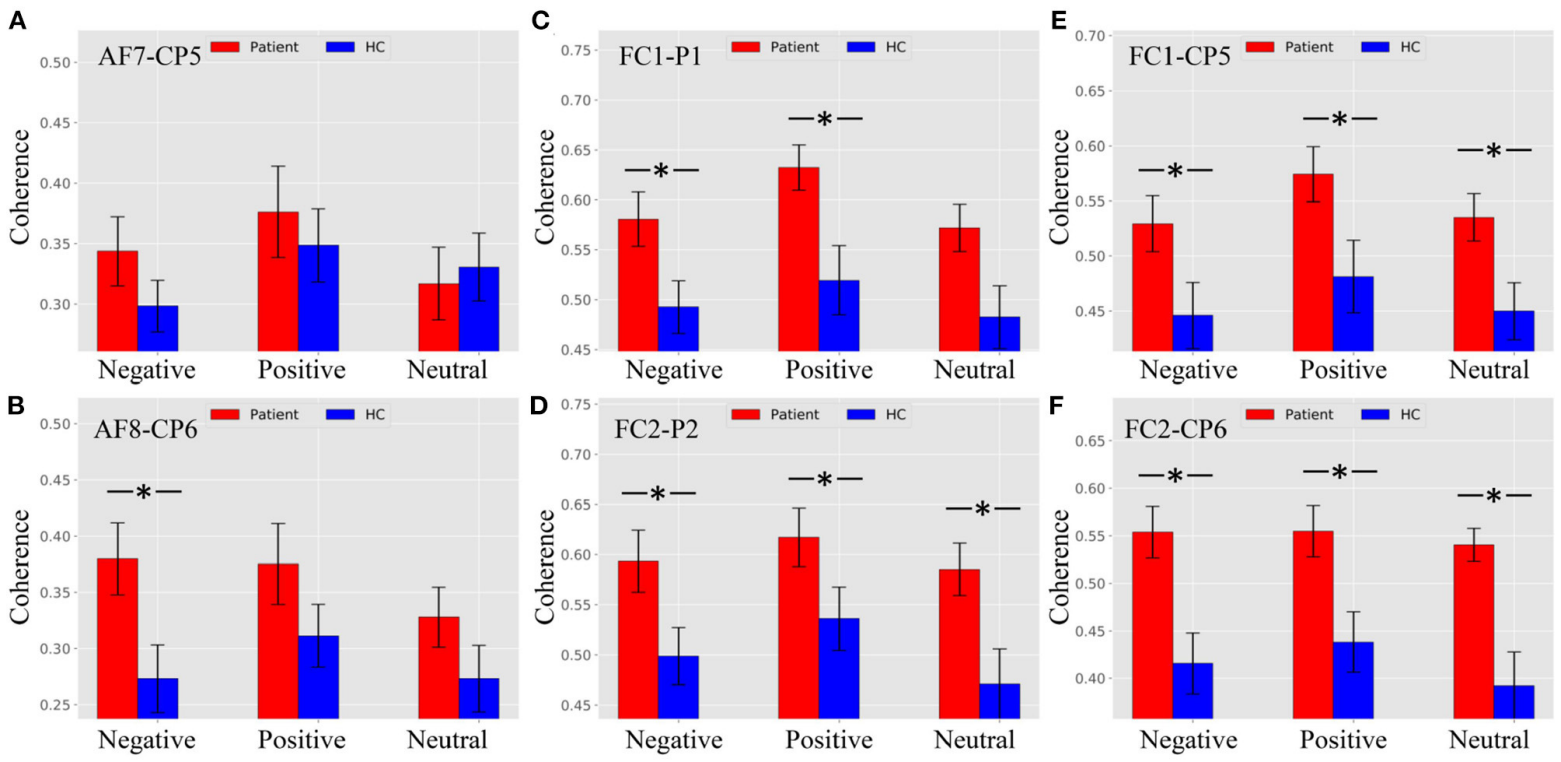
Alpha ERPCoh between frontal and parietal electrode within 180–280 ms. **(A)** Alpha ERPCoh between patients and HCs for all stimulus trials at the AF7–CP5 electrode pair. **(B)** Alpha ERPCoh between patients and HCs for all stimulus trials at the AF8–CP6 electrode pair. **(C)** Alpha ERPCoh between patients and HCs for all stimulus trials at the FC1–P1 electrode pair. **(D)** Alpha ERPCoh between patients and HCs for all stimulus trials at the FC2–P2 electrode pair. **(E)** Alpha ERPCoh between patients and HCs for all stimulus trials at the FC1–CP5 electrode pair. **(F)** Alpha ERPCoh between patients and HCs for all stimulus trials at the FC2–CP6 electrode pair. *p_*FDR*−*corrected*_ ≤ 0.0367.

Figure 5A shows that there was no difference between patients and HCs for all stimulus trials at the AF7–CP5 electrode pair. As shown in Figure 5B, the alpha ERPCoh in the patients was larger than in HCs for negative stimulus trials at the AF8–CP6 electrode pair (negative: *p* = 0.0116). However, the alpha coherence of the AF8–CP6 electrode pair for negative and neutral stimulus trails approached marginal significance (positive: *p* = 0.0902; neutral: *p* = 0.0942).

Figures 5C–F show the same pattern that the alpha band ERPCoh in patients was significantly larger than in HCs at the FC1–P1, FC2–P2, FC1–CP5, and FC2–CP6 electrode pairs.

**Figure 6:**
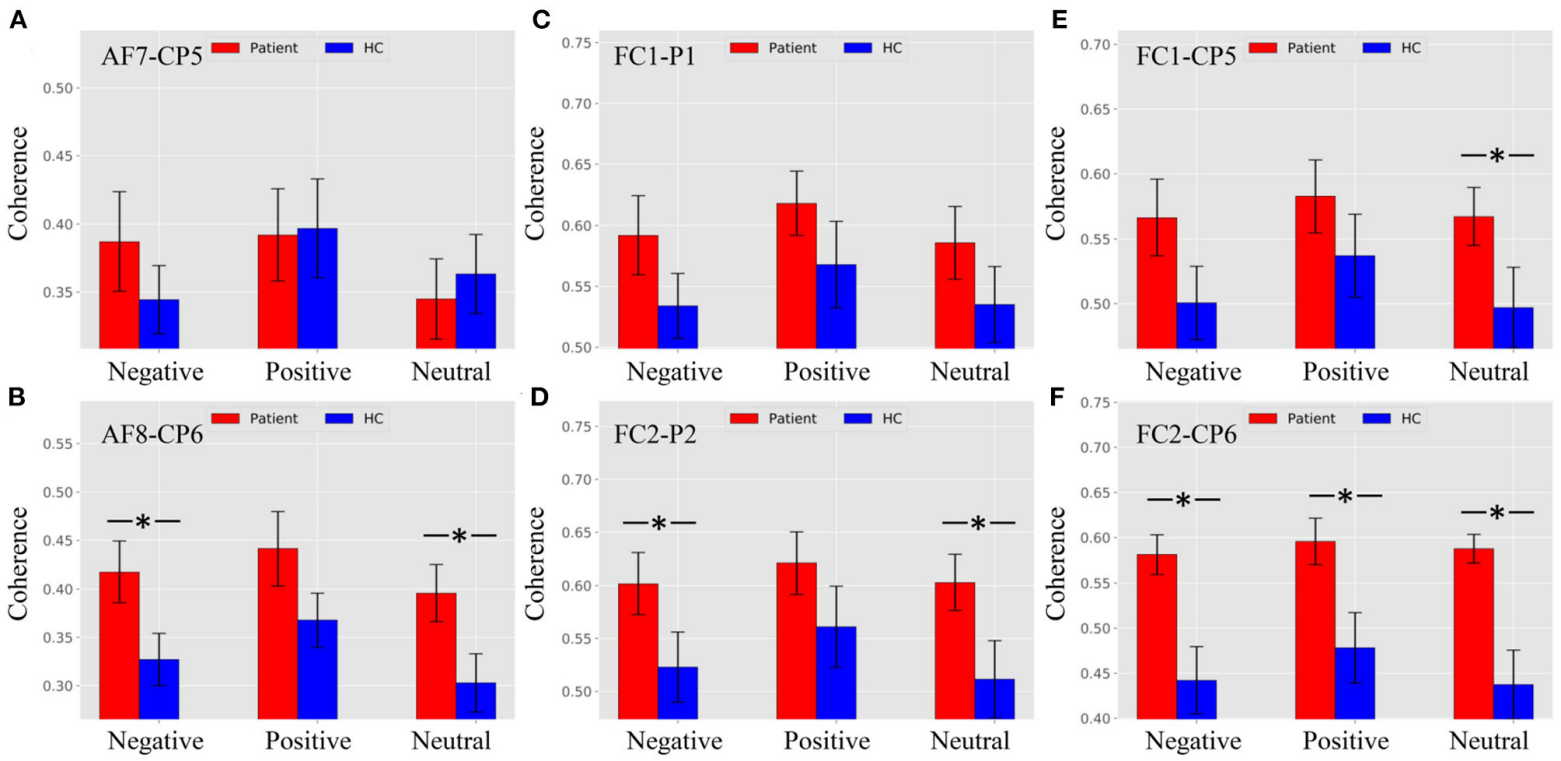
Theta ERPCoh between the frontal and parietal regions in patients and healthy controls (HCs) under different emotional stimuli in ROI (the time window ofnP200, theta band). **(A)** Theta ERPCoh between patients and HCs for all stimulus trials at the AF7–CP5 electrode pair. **(B)** Theta ERPCoh between patients and HCs for all stimulus trials at the AF8–CP6 electrode pair. **(C)** Theta ERPCoh between patients and HCs for all stimulus trials at the FC1–P1 electrode pair. **(D)** Theta ERPCoh between patients and HCs for all stimulus trials at the FC2–P2 electrode pair. **(E)** Theta ERPCoh between patients and HCs for all stimulus trials at the FC1–CP5 electrode pair. **(F)** Theta ERPCoh between patients and HCs for all stimulus trials at the FC2–CP6 electrode pair. *p_*FDR*−*corrected*_ ≤ 0.0454.

As shown in Figure 6A, there were no significant differences between patients and HCs at the AF8–CP6 electrode pair. Figure 6B shows that the theta ERPCoh in patients was larger than in HCs for negative and neutral trials, while no difference was found in positive trials at the AF8–CP6 electrode pair (negative: *p* = 0.0116; neutral: *p* = 0.0094; positive: *p* = 0.0902).

As shown in Figure 6C, there were no significant differences between patients and HCs at the FC1–P1 electrode pair. Figure 6D shows that the theta band ERPCoh in patients was larger than in HCs for negative and neutral trials, while no difference was found in positive trials at the FC2–P2 electrode pair (negative: *p* = 0.0454; neutral: *p* = 0.0276; positive: *p* = 0.1168).

As shown in Figure 6E, the theta ERPCoh in the patients was larger than in HCs for neutral stimulus trials at the FC1–CP5 electrode pair (negative: *p* = 0.0404). However, there was no difference for negative and neutral stimulus trials in theta ERPCoh of the AF7–CP5 electrode pair (negative: *p* = 0.0623; positive: *p* = 0.1513). Figure 6F showed that the theta ERPCoh in patients was significantly larger than in HCs at the FC2–CP6 electrode pair (negative: *p* = 0.0016; positive: *p* = 0.0095; neutral: *p* = 0.0005).

### Correlation Between Prolactin and Event-Related Phase Coherence

Under negative and positive stimuli, we inspected a significant pattern that the alpha band ERPCoh was significantly correlated with the PRL in patients at the AF7–CP5, AF8–CP6, FC1–P1, FC2–P2, FC2–CP5, and FC2–CP6 electrode pairs (see Table 2).

**Table 2:** Correlations between frontoparietal alpha event-related phase coherence with prolactin in prolactinomas.

	**Stimulus**	**Person_** ***r***	***p***		**Stimulus**	**Person_** ***r***	***p***
AF7–CP5	Negative	**0.61**	**0.0045**	AF8–CP6	Negative	**0.56**	**0.01**
	Positive	**0.57**	**0.0082**		Positive	**0.47**	**0.038**
	Neutral	**0.48**	**0.01**		Neutral	0.17	0.48
FC1–P1	Negative	**0.63**	**0.0031**	FC2–P2	Negative	**0.69**	**0.00071**
	Positive	**0.63**	**0.0032**		Positive	**0.58**	**0.0077**
	Neutral	0.28	0.24		Neutral	**0.47**	**0.035**
FC1–CP5	Negative	**0.65**	**0.0019**	FC2–CP6	Negative	**0.73**	**0.00024**
	Positive	**0.49**	**0.029**		Positive	**0.58**	**0.0076**
	Neutral	0.39	0.089		Neutral	**0.64**	**0.0026**

*Bold values mean significant correlation between Prolactin level and ERPCoh*.

Under neutral stimuli, a significant correlation was also found at the AF7–CP5, FC2–P2, and FC2–CP6 electrode pairs, while there was no correlation at the AF8–CP6, FC1–P1, and FC1–CP5 electrode pairs (see Table 2).

As for the correlations between frontoparietal theta ERPCoh with PRL in patients, a positive correlation has also been found in the right-lateralized frontoparietal network, including FC2–P2 (negative: *r* = 0.55, *p* = 0.012; positive: *r* = 0.52, *p* = 0.017) and FC2–CP6 (negative: *r* = 0.55, *p* = 0.011; positive: *r* = 0.44, *p* = 0.052).

## Discussion

The current study is the first to demonstrate the altered attention network in prolactinomas. The findings showed the correlation between frontoparietal coherence and endogenous hormone levels in prolactinomas. As for frontoparietal ERPCoh, patients displayed enhanced alpha and theta ERPCoh compared with the HCs, especially in the right-lateralized hemisphere, implying changes within the frontoparietal network. Furthermore, the frontoparietal ERPCoh was positively correlated with PRL, indicating influences of endogenous hormones on brain compensation.

Prolactinomas may have difficulties in attracting attention resources rapidly and automatically. Hence, prolactinomas may decrease processing efficiency, but we postulate that prolactinomas can compensate for the lower efficiency through increased connectivity of the frontoparietal network. Increased connectivity may come from the compensatory mechanisms of the brain; however, to some extent, the compensation ability will decrease if the structural and functional connectivity is still progressively impaired due to the abnormal hormone levels or tumor compression. Our team previously found that increased thalamocortical and cerebellar–cerebral functional connectivity (FC) was associated with endogenous hormone levels, which supports a functional compensatory mechanism that occurs before the cascade of structural damage (43). Yao et al. (43) found that prolactinoma patients showed increased FC mostly between the posterior brain regions and temporal lobes, namely, the cerebellum, precuneus, posterior cingulate cortex (PCC), and bilateral temporal fusiform cortex (TFusC). As a result, enhanced connections of posterior brain regions in these patients might be used as an imaging biomarker for cognitive dysfunctions (44). Although these prolactinomas in the research of Yao showed increased FC between these brain regions, there are limitations in dictating whether these patients suffer from the dysfunctions of attention processing due to the absence of using tasks that require attention processing. Therefore, combined with the results of the present experiment, it could be initially considered that pituitary patients may have attention processing impairments. Besides, attentional control particularly activates the right hemisphere, including ventral frontal cortices and temporoparietal junction (45). This research supported the existence of a right-lateralized frontoparietal network that is involved in directing attention to a stimulus. A general frontoparietal network in the right hemisphere has been reported to be related to orienting and maintaining attention to a new stimulus (46). In humans, there is a denser concentration of noradrenaline in the right than in the left thalamus, which might be related to the right lateralization of the frontoparietal network (47). Therefore, we speculate that if the frontoparietal network, especially the right-lateralized one, is damaged, the coherence between frontal and parietal regions may be enhanced to complete basic cognitive functions. This phenomenon may reflect compensatory activity within the frontoparietal network. The compensatory mechanism above is beneficial in maintaining the normal physiological function, which has been regarded as a common phenomenon in some psychiatric diseases (48–50).

We found a significant pattern that the alpha and theta ERPCoh between frontoparietal regions were positively correlated with the PRL in patients. Previous research reported that PRL overproduction could lead to cognitive impairments (51). Furthermore, the overproduction of PRL has been found to impair the efficacy of cognitive processing via the dopamine pathway, which was altered in prolactinomas due to the anti-correlation between PRL release and dopamine production (52). Biologically, the overproduction of PRL could have negative effects on myelin oligodendrocyte glycoprotein via enhancing the number of cells secreting antibodies, and then impair neuronal changes and plasticity (53). Expression of PRL is widely distributed in the cerebral cortex, thalamus, hypothalamus, amygdala, etc. (54). Besides, it has been reported that testosterone exerts neuroprotective effects on structural and functional connections by allowing actin cytoskeleton involvement (55). Since testosterone level in serum can be suppressed by excess PRL in serum due to prolactinomas, the lower testosterone level may also induce the impaired connectivity in the brain. Therefore, patients with neuroendocrine tumors may have cognitive impairments because of their abnormal hormone levels. Our team has demonstrated that higher PRL levels are correlated with worse cognitive function (6–8, 56). Yao et al. (13) revealed that prolactinomas demonstrated a gray matter volume (GMV) decrease in the prefrontal cortex, reflecting that abnormally elevated PRL levels have a detrimental influence on the cortex relevant to attention processing. Our interesting findings on the hyperactive coherence within the frontoparietal networks may be one of the potential pathophysiological factors that impair attention processing impairments in prolactinomas. Electrophysiological findings in this research were almost consistent with previous magnetic resonance imaging (MRI) studies by showing that significantly increased functional connectivity between distinct brain regions and endogenous hormone levels were also positively correlated with increased functional connectivity (43). Hence, abnormally high PRL levels exert negative effects on brain structures, then leading to increased frontoparietal coherence. These findings demonstrated the significance of endogenous hormones for functional compensation in prolactinomas.

There were multiple limitations discussed in this research. The present study cannot precisely figure out which neuroanatomical regions are damaged in prolactinomas because of the spatial limitation of scalp EEG. Thus, combining neuroimaging techniques, such as structure MRI and fMRI, is promising in the future to map more detailed neural circuits, which may be potentially impaired in prolactinomas (57).

## Conclusions

Overall, alterations in frontoparietal coherence linked to attention processing between prolactinomas and HCs have been observed. Our findings demonstrated increased frontoparietal coherence in prolactinomas, especially in the right-lateralized hemisphere. Importantly, the frontoparietal coherence was positively correlated with altered endogenous hormone levels, implying the significance of PRL for adaptive brain compensation in prolactinomas. Thus, the altered frontoparietal coherence as electrophysiological features may potentially predict the impaired attention processing in prolactinomas.

## Data Availability Statement

The raw data supporting the conclusions of this article will be made available by the authors, without undue reservation.

## Ethics Statement

All procedures followed the Declaration of Helsinki and were approved by the Ethical Committee of Wuhan School of Clinical Medicine, Southern Medical University (China). The number of the approved ethical statement is [2014] 024-1. The patients/participants provided their written informed consent to participate in this study.

## Author Contributions

GX and JS were responsible for the study concept and design. CC, AC, and JL collected the EEG data. CC and YW assisted with data analysis and interpretation of findings, drafted the manuscript, and contributed to the interpretation and manuscript revision. CC performed the statistical analysis. JL critically revised the manuscript and results interpretation. All authors have reviewed the content and approved the final version for publication.

## Conflict of Interest

The authors declare that the research was conducted in the absence of any commercial or financial relationships that could be construed as a potential conflict of interest.

## Publisher's Note

All claims expressed in this article are solely those of the authors and do not necessarily represent those of their affiliated organizations, or those of the publisher, the editors and the reviewers. Any product that may be evaluated in this article, or claim that may be made by its manufacturer, is not guaranteed or endorsed by the publisher.
