# Supplementary figures and images for: Altered Connectivity of the Frontoparietal Network During Attention Processing in Prolactinomas
Source: Front Neurol. 2021 Aug 30;12:638851. doi: 10.3389/fneur.2021.638851 (PMC8435841; doi:10.3389/fneur.2021.638851)

**Patient 1**

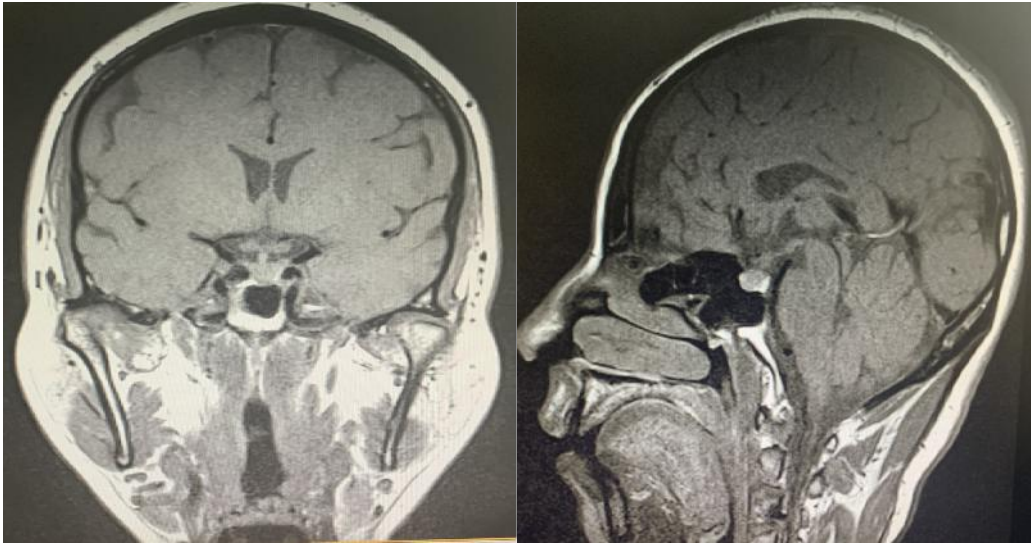

**Patient 2**

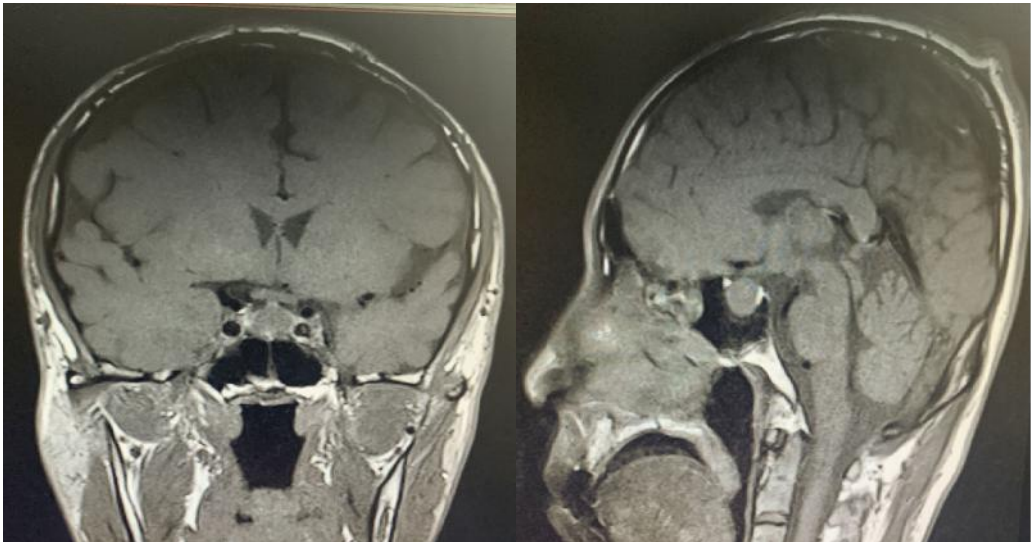

**Patient 3**

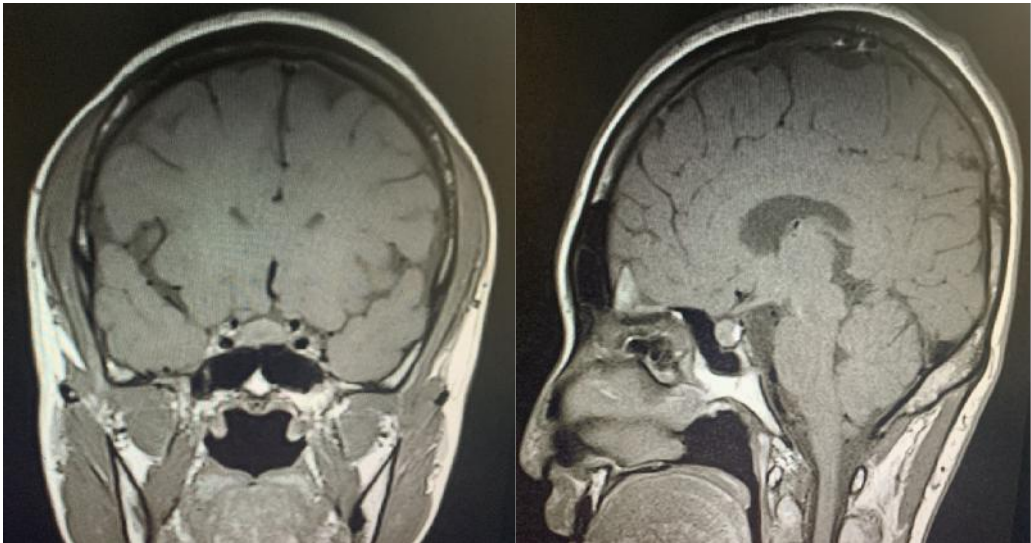

Patient 4

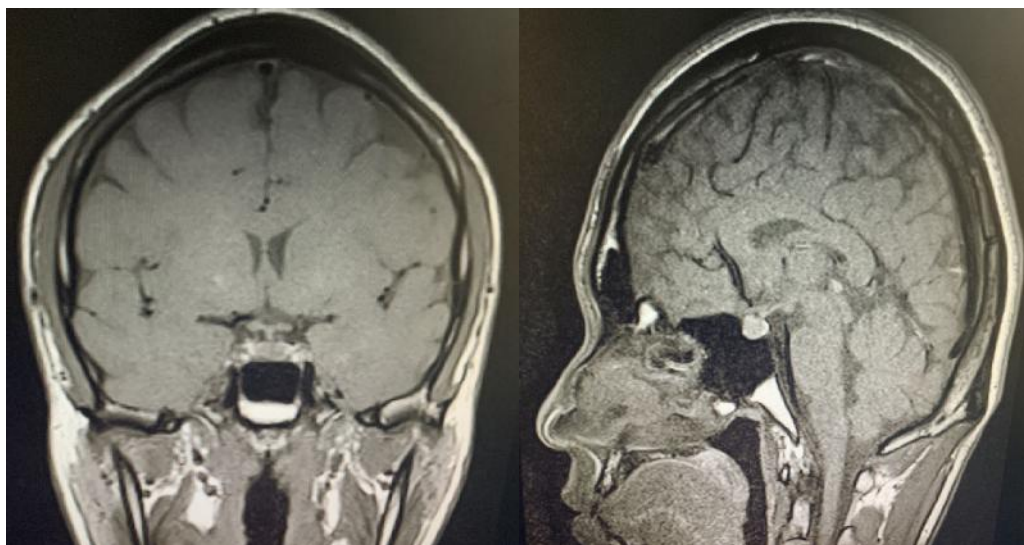

Supplement: Supplementary file 1 [file Data_Sheet_1.PDF]
