# Supplementary material for: Altered Connectivity of the Frontoparietal Network During Attention Processing in Prolactinomas
Source: Front Neurol. 2021 Aug 30;12:638851. doi: 10.3389/fneur.2021.638851 (PMC8435841; doi:10.3389/fneur.2021.638851)
Supplement: Supplementary file 2 [file Table_1.doc]

**Table.1.**Means and standard deviations for arousal and valence for each IAPS

**Mean valence and IAPS**

**arousal ratings Positive Neutral Negative P**

| **Valence** | 6.1540±1.7842 | 4.9400±0.1812 | 3.188±0.6594 | ＜0.01 |
| --- | --- | --- | --- | --- |
| **Arousal** | 5.4160±0.3135^a^ | 2.5673±0.5062 | 5.4887±0.3905^a^ | ＜0.01 |

a：*P*<0.05，compared with neutral stimuli;
